# Limited Promiscuity of HLA-DRB1 Presented Peptides Derived of Blood Coagulation Factor VIII 

**DOI:** 10.1371/journal.pone.0080239

**Published:** 2013-11-14

**Authors:** Simon D. van Haren, Aleksandra Wroblewska, Eszter Herczenik, Paul H. Kaijen, Aleksandra Ruminska, Anja ten Brinke, Alexander B. Meijer, Jan Voorberg

**Affiliations:** 1 Department of Plasma Proteins, Sanquin-AMC Landsteiner and Van Creveld Laboratory, Amsterdam, The Netherlands; 2 Department of Immunopathology, Sanquin Research, Amsterdam, The Netherlands; 3 Utrecht Institute for Pharmaceutical Sciences, Utrecht University, Utrecht, The Netherlands; National Institute of Infectious Diseases, Japan

## Abstract

The formation of inhibitory antibodies directed against coagulation factor VIII (FVIII) is a severe complication in the treatment of hemophilia A patients. The induction of anti-FVIII antibodies is a CD4^+^ T cell-dependent process. Activation of FVIII-specific CD4^+^ T cells is dependent on the presentation of FVIII-derived peptides on MHC class II by antigen-presenting cells. Previously, we have shown that FVIII-pulsed human monocyte-derived dendritic cells can present peptides from several FVIII domains. In this study we show that FVIII peptides are presented on immature as well as mature dendritic cells. In immature dendritic cells half of the FVIII-loaded MHC class II molecules are retained within the cell, whereas in LPS-matured dendritic cells the majority of MHC class II/peptide complexes is present on the plasma membrane. Time-course studies revealed that presentation of FVIII-derived peptides was optimal between 12 and 24 hours after maturation but persisted for at least 96 hours. We also show that macrophages are able to internalize FVIII as efficiently as dendritic cells, however FVIII was presented on MHC class II with a lower efficiency and with different epitopes compared to dendritic cells. In total, 48 FVIII core-peptides were identified using a DCs derived of 8 different donors. Five HLA-promiscuous FVIII peptide regions were found – these were presented by at least 4 out of 8 donors. The remaining 42 peptide core regions in FVIII were presented by DCs derived from a single (30 peptides) or two to three donors (12 peptides). Overall, our findings show that a broad repertoire of FVIII peptides can be presented on HLA-DR.

## Introduction

Professional antigen-presenting cells (APC) such as dendritic cells (DCs) and macrophages are able to process antigens and present antigen-derived peptides in the context of major histocompatibility complex (MHC) molecules. Presentation of internalized antigens on MHC class II by dendritic cells is required for the activation of CD4^+^ T helper cells, which subsequently can stimulate B cells to produce high-affinity antibodies. Activation of CD4^+^ T cells depends on recognition of a specific antigen-derived peptide in context of appropriate MHC II complex but also requires maturation of dendritic cells leading to upregulation of co-stimulatory molecules such as CD40, CD80 and CD86 resulting in the release of specific cytokines that determine T cell differentiation into various lineages which include Th1, Th2, Th17 or regulatory T cells. Th1, Th2 and Th17 cells have been shown to contribute to FVIII-specific immune responses in hemophilia A [1,2]. Re-stimulation of memory CD4^+^ T cells does not require upregulation of co-stimulatory molecules and can also occur following presentation of FVIII peptides on macrophages and FVIII-specific B cells. The diversity of CD4^+^ T cell responses is dependent both on the repertoire of CD4^+^ T cells and on the peptide-binding properties of MHC molecules present on APC.

Due to the unique properties of each MHC class II allele in their ability to present different antigen-derived peptides, some alleles are considered to be associated with the etiology of autoimmune disorders or with undesired antibody responses towards protein therapeutics [3-5]. Moreover, several MHC class I and class II alleles are linked with susceptibility to and outcome of viral infections, such as human immunodeficiency virus type 1, hepatitis C virus and SARS-coronavirus [6,7]. The identification of naturally presented peptides of clinically relevant antigens can help to further improve vaccine design as well as the identification of pathogenic T cells involved in auto-immunity or immune responses to therapeutic proteins. 

Previously, we have reported that it is feasible to probe the repertoire of MHC class II-presented peptides using APC pulsed with model-antigen coagulation factor VIII (FVIII) [8]. FVIII can be efficiently internalized by APCs both in vitro as well as in vivo [9,10]. In hemophilia A patients, which have reduced or diminished endogenous FVIII levels in the circulation, replacement therapy with recombinant or plasma-derived FVIII can lead to the formation of FVIII-neutralizing antibodies, commonly referred to as “inhibitors”. It has been suggested that the endocytosis of FVIII is receptor-mediated [9] and there is evidence that FVIII is efficiently presented on MHC class II [8,9]. Processing of FVIII by APCs leads to the presentation of various FVIII peptides, which are derived from different domains present in the molecule [8]. As a result, CD4^+^ T-cell responses in patients with severe hemophilia A are of polyclonal origin and directed against multiple epitopes [11-13]. Generated peptides are either promiscuously presented on multiple MHC class II molecules or their presentation is unique and MHC-haplotype restricted [8]. 

In this study we show using ovalbumin and FVIII that several distinct regions can be presented on MHC class II. We also show that FVIII-derived peptides are presented for extended periods of time. FVIII-pulsed macrophages and DCs derived of the same donor present different sets of peptides on MHC class II. Analysis of a panels of DCs derived of multiple donors indicate that only a limited number of FVIII peptides is presented in a promiscuous manner. 

## Methods

### Ethics statement

Blood was drawn from HLA-typed healthy anonymous volunteers with informed, written consent in accordance with Dutch regulations and following approval from Sanquin Ethical Advisory Board in accordance with the Declaration of Helsinki. The volunteers nor those taking the samples know for what project specific samples were used. Moreover, allowed annual sample volume and frequency of donation were established after consultation with Sanquin Medical Secretary. Additional files, such as form signed by blood donors, are attached. The named institutional review board specifically approved this study. 

### Reagents

In this study, the following reagents were used: Recombinant human FVIII (Advate) was kindly provided by Dr. B.M. Reipert (Baxter Healthcare Corporation, Vienna, Austria). EndoGrade® ovalbumin was from Hyglos (Bernried, Germany). CD14 microbeads (MACS, Miltenyi Biotech Bergisch Gladbach, Germany), anti-CD80-FITC, anti-CD83-APC, anti-CD86-APC (BD Biosciences, USA), mouse-anti-ovalbumin (St. Louis, USA) and anti-CD14-PE (Sanquin Reagents, Amsterdam, the Netherlands). Cellgro DC serum-free medium, IL-4, M-CSF and GM-CSF were obtained from CellGenix (Freiburg, Germany). LPS was obtained from Sigma-Aldrich (St. Louis, USA). Hybridomas L243 and IVA-12 (anti-HLA-DR) were obtained from ATCC (Wesel, Germany). 

### FVIII endocytosis by monocyte-derived DCs and macrophages

Human monocyte-derived DCs (moDCs) and macrophages were prepared as described previously [14,15]. After 5 days of culture, the immature moDCs or macrophages were washed and replated in Cellgro medium supplemented with either GM-CSF and IL-4 or M-CSF at a concentration of 5∙10^6^ cells/ml in a final volume of 1 ml. Cells were incubated with 100 nM FVIII or 100 µg/ml ovalbumin for 5h prior to induction of maturation. After 5h, the immature moDCs or macrophages were maturated using 1 μg/ml LPS for 24 h in the presence of 1% human serum. The adherent maturated moDCs were detached by 5 minute incubation with phosphate buffered saline (PBS) containing 0.25% trisodiumcitrate and washed before analysis.

### Flow cytometric analysis of FVIII endocytosis by monocyte-derived DCs and macrophages

Endocytosis of FVIII was measured as described previously [15]. Briefly, FVIII endocytosis was performed as described above. After endocytosis, cells were washed once with cold TRIS-buffered saline (TBS) and fixed with 1% paraformaldehyde (Polysciences, Eppelheim, Germany). Cell-associated and intracellular FVIII was subsequently measured by incubation of the cells with FITC-conjugated anti-FVIII monoclonal antibody CLB-Cag117 in TBS with 0.05% saponin and 0.5% human serum albumin. Mean Fluorescence Intensity (MFI) was measured using an LSR II flow cytometer (BD Biosciences, Uppsala, Sweden).

### Flow cytometric analysis of cell-surface phenotype

For determination of the phenotype, cells were washed with PBS containing 0.5% bovine serum albumin (PBS/0.5%BSA) and incubated with 50 μl 1 μg/ml antibody or appropriate isotype controls diluted in PBS/0.5%BSA for 30 min at 4°C. Cells were washed twice and resuspended in PBS/0.5%BSA. Cells were analyzed on an LSRII flow cytometer (Beckton Dickinson, San Jose, USA) and analyzed with Flowjo software version 7.5.5 (Tree Star Inc., Ashland, USA).

### Purification of HLA-DR presented peptides on moDCs

HLA-DR molecules were purified from FVIII-treated, maturated moDCs or macrophages essentially as described previously[8]. Briefly, cell pellets were lysed using 50 mM Tris pH 7.0 containing 4% Igepal CA-630 (Sigma, St. Louis, USA). HLA-DR was purified from the detergent-soluble fraction by immunoaffinity chromatography using antibody L243-coupled to CNBr Sepharose 4B (Amersham Biosciences, Buckinghamshire, UK) in the presence of protease inhibitors (Complete Protease Inhibitor Cocktail Tablet, 1 tablet per 50 ml buffer, Roche Diagnostics GmbH, Mannheim, Germany). After washing the Sepharose 5 times with 50 mM Tris-HCl pH 7.0, peptides were acid eluted with 10% acetic acid for 15 minutes at 70°C. Eluted peptides were purified from the acetic acid eluate using a C18 ziptip (Millipore, Billerica, USA). 

### Subcellular fractionation

After endocytosis of FVIII or ovalbumin, cells were homogenized in STE buffer (1.25 M sucrose, 100 mM Tris, 5 mM EDTA) containing protease inhibitors (Complete Protease Inhibitor Cocktail Tablet, 1 tablet per 50 ml buffer, Roche Diagnostics GmbH, Mannheim, Germany). Postnuclear supernatants (PNS) were obtained by centrifugation at 150xg for 5 minutes at 4°C and loaded on a linear sucrose gradient (5–50%, 11 ml). Density gradients were obtained by centrifugation for 90 min at 200.000xg at 4° C in a Beckmann Optima^TM^ L-100 XP ultracentrifuge (Beckmann Instruments, Palo Alto, CA, USA). Fractions (0.5 ml) were collected from high to low sucrose density and subsequently analyzed for relative MHC class II protein content. Nunc-Maxisorp 96-well plates were coated with anti HLA-DR antibody IVA-12 (5 μg/ml) in carbonate buffer (50 mM NaHCO_3_, pH 9.8) overnight at 4°C. Fractions were subsequently diluted in PBS/0.1%Tween 20/0.3% BSA and added to the plate for 1h at 37°C. MHC class II was detected with HRP-labeled antibody L243.

### Tryptic digestion of proteins

The HLA-content of each fraction was measured by ELISA. Fractions that corresponded to peaks in HLA-DR content were pooled as indicated. Ninety percent of the pools was analyzed for HLA-DR presented peptides and the remaining 10% for total protein content by tryptic digestion. Samples (50 μL) were boiled at 100°C for 5 minutes to break organelles. 6 M Urea (20 μL) was added and incubated at RT for 15 minutes. Next, urea concentration was reduced to 1 M by addition of 50 mM ammonium bicarbonate buffer. Subsequently, dithiothreitol (DTT) was added to a final concentration of 7.7 mM to reduced disulfide bonds. Samples were incubated for 30 minutes at RT. Next, iodoacetamide was added to a final concentration of 8.9 mM and samples were incubated for 30 minutes at RT in the dark. Finally, samples were digested with 6.25 ng/ml trypsin overnight at 37°C. Peptides were prepared for mass spectrometry analysis by purification using a C18 ziptip (Millipore, Billerica, USA).

### Peptide identification by mass spectrometry

The identification of peptides was performed essentially as described previously[8]. Briefly, Eluted peptides were separated using a reversed-phase C18 column at a flowrate of 100 nl/min with gradient from 0% to 35% (v/v) acetonitrile with 0.1% HAc. Eluted peptides were sprayed directly into the LTQ Orbitrap XL mass spectrometer (Thermo Fisher Scientific, Bremen, Germany) using a nanoelectrospray source with a spray voltage of 1.9 kV. Collision induced dissociation in the ion-trap (35% normalized collision energy) for the five most intensive precursor ions selected from each full scan in the Orbitrap (300-2000 m/z, resolving power 30.000) was performed. To obtain a high mass accuracy, the LTQ Orbitrap was calibrated on a monthly basis using a calibration solution consisting of caffeine, MRFA and Ultramark 1621 as recommended by the manufacturer. Peptides were identified using a Sequest search algorithm against UniprotKB non-redundant protein database 25.H_sapiens.fasta (53,784 non-redundant entries actually searched), utilizing Proteome Discoverer release version 1.1 software (Thermo Scientific, Bremen, Germany) [16]. For trypsin-digested proteins, which were treated with iodoacetamide, searches were performed with a static carbamidomethyl modification. Identification of peptides was performed using the following filter settings. During the Sequest search, we allowed a mass deviation of 20 ppm. Fragment mass tolerance was 0.8 Da. For semi-quantitative analysis of MHC class II bound peptides by differential expression analysis, duplicate endocytosis experiments were analyzed with SIEVE™ release version 1.2.0 software (Thermo Scientific, Bremen, Germany). This software was used to compare the duplicate experiments and subsequently analyze differences in peptide abundance between samples incubated under different maturation conditions. For peptide identification, the same criteria were used as described above.

## Results

### FVIII peptides are presented on immature as well as mature DCs

**Figure 1:**
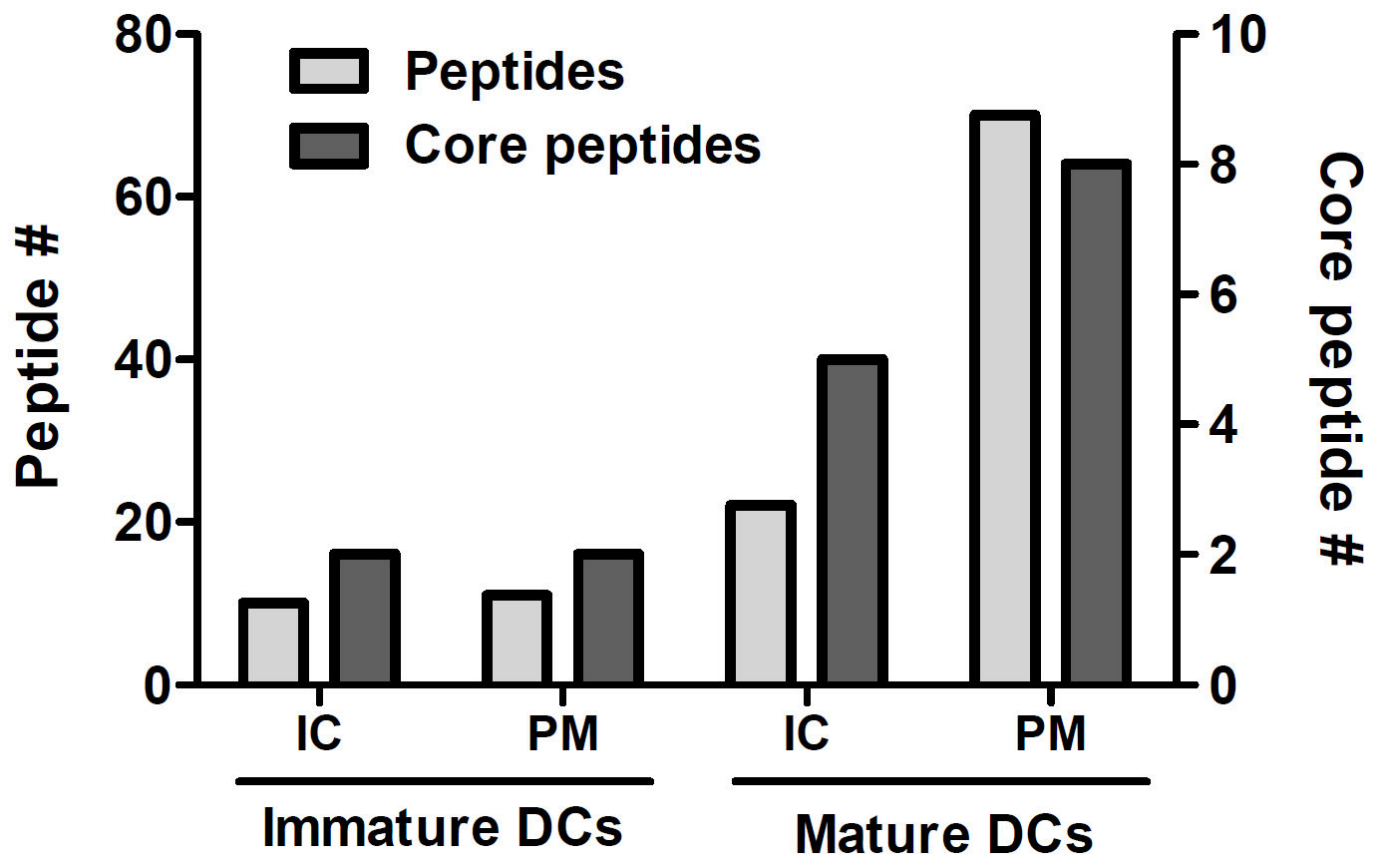
Presentation of FVIII peptides on immature and mature DCs. The presentation of FVIII peptides was compared between immature DCs and LPS-matured DCs. Cells were homogenized and PNS was fractionated on a sucrose density-gradient. Fractions from the sucrose gradient were analyzed by ELISA for MHC class II molecules and divided into a plasma membrane pool (PM) and an intracellular pool (IC) (see Figure S2 in File S1). Peptides were purified from the pools using L243-sepharose and analyzed by mass spectrometry. On the left y-axis the total number of FVIII peptides recovered from MHC class II is indicated (Peptide #). On the right y-axis number of “core peptides” (Core peptide #) is indicated. Core peptides are defined as a set of peptides with an overlapping sequence harboring an MHC class II binding motif. Heterogeneity in peptide length is due to amino-terminal and/or carboxy-terminal trimming of the presented peptides.

We have previously shown that FVIII pulsed monocyte-derived dendritic cells (moDCs) efficiently present FVIII-derived peptides on MHC class II [8] . To gain more insight into intracellular fate of FVIII peptides loaded on MHC class II molecules, subcellular fractionations were performed using a sucrose-density gradient to compare the amount of MHC class II-bound FVIII peptides present inside the cells with that actually presented on the plasma membrane. Per condition, 15 million moDCs were used. Sucrose gradients of immature and mature DCs comprise 3 distinct peaks of MHC class II-containing fractions, which were pooled into 3 different pools (Figure S1A in File S1). Tryptic digestion and subsequent mass-spectrometry analysis (Figure S1B in File S1) suggests that the pool corresponding to the highest sucrose density (pool 1) contains mostly intracellular proteins derived from organelles such as mitochondria, MHC class II loading compartments and other vesicles. The second pool is enriched in plasma membrane-associated components. The top fractions (pool 3), which are the lowest in sucrose density, contain material from cell organelles that lysed during the cell disruption procedure. Therefore, these fractions were excluded from subsequent analysis. HLA-DR from the first (intracellular) and the second (plasma membrane) pool was subsequently purified and analyzed for the presence of FVIII peptides. Immature DCs contain a significant amount of FVIII peptides, which have been processed and loaded on HLA-DR (Figure 1). The amount of these complexes is highly similar between pool 1, containing 10 FVIII derived peptides, and pool 2, containing 11 FVIII derived peptides. This suggests that approximately half of these complexes is already presented on the plasma membrane of immature DCs. DCs maturated with LPS presented a total of 92 FVIII peptides, the majority of which (70) were localized on the plasma membrane. Together, these data show that mature DCs are 2-3 times more efficient in presentation of FVIII peptides when compared to immature DCs. 

**Figure 2:**
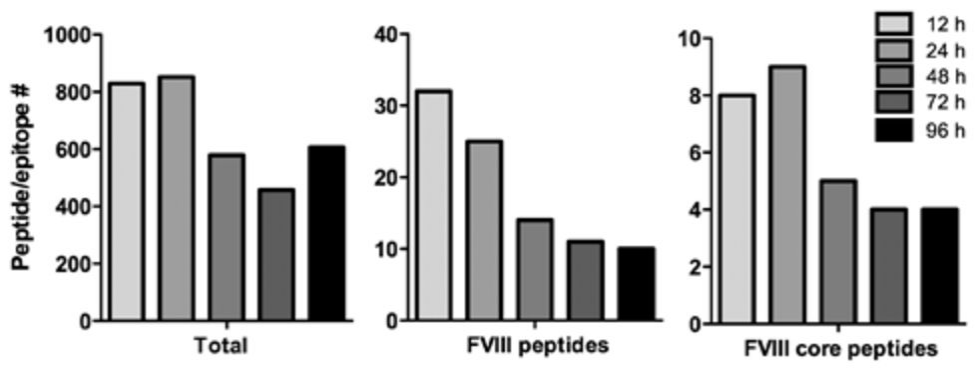
Persistent presentation on FVIII peptides on mature DCs. Endocytosis of FVIII by immature DCs was followed by maturation with LPS for either 12h, 24h, 48h, 72h or 96h. HLA-DRB1 bound peptides were purified from L243-sepharose and analyzed by mass spectrometry. Bar diagrams indicate the total amount of DRB1-bound peptides identified under each condition as well as the amount of FVIII peptides and core peptides. Core peptides are defined as a set of peptides with an overlapping sequence harboring an MHC class II binding motif. Heterogeneity in peptide length is due to amino-terminal and/or carboxy-terminal trimming of the presented peptides.

**Figure 3:**
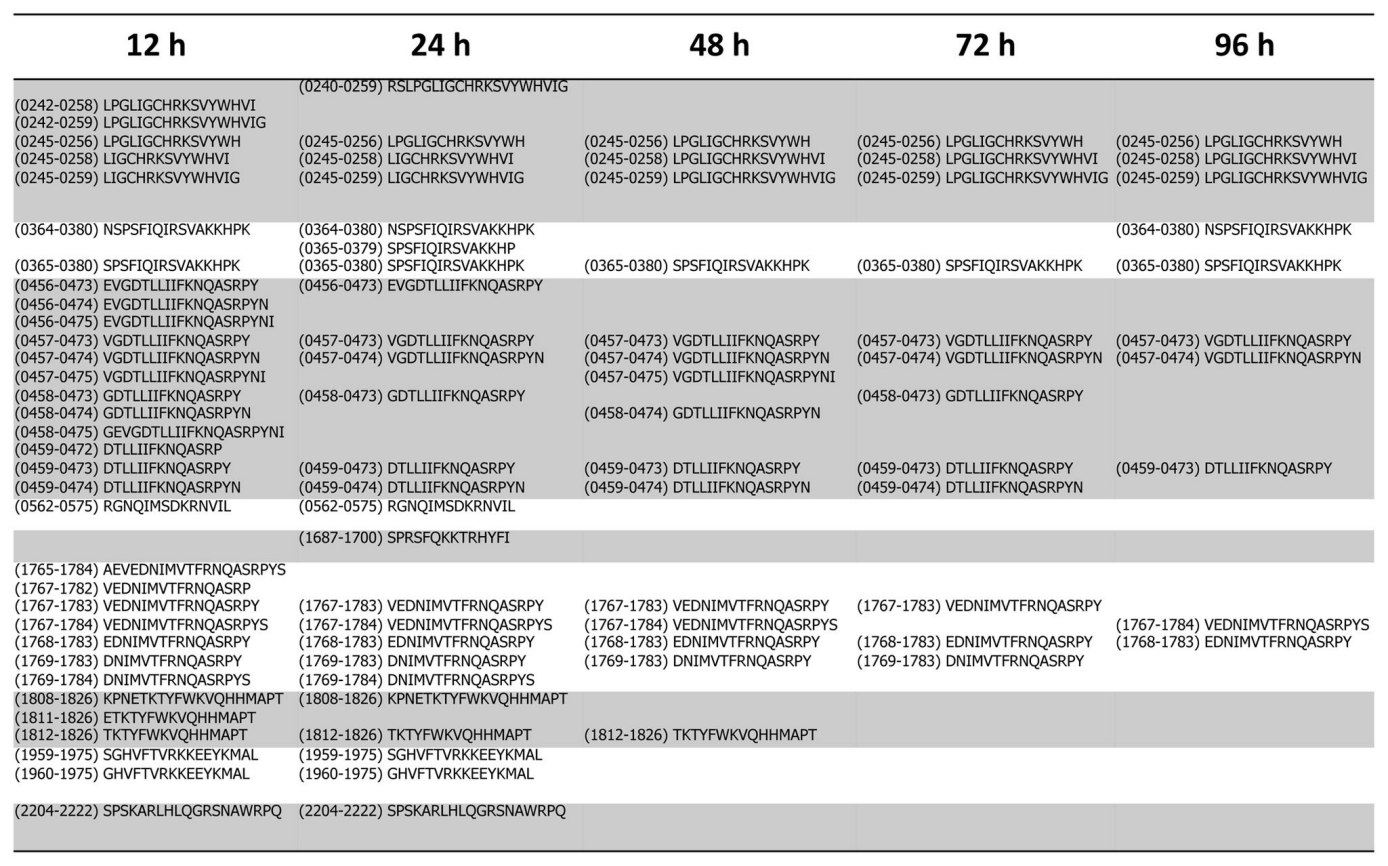
Graphical representation of all FVIII peptides presented at each time point.

We also addressed the persistence of peptide-MHC class II complexes on mature DCs (Figure 2). Presentation of FVIII peptides was optimal between 12 and 24 hours. However, approximately half of the FVIII peptides remains presented on MHC class II for at least up to 96 hours (Figure 2, later time points were not included in current study). Sequence of the FVIII peptides presented after 96 hours corresponds to that of peptides presented at earlier time points (Figure 3), which suggests that either presentation of individual peptides persists for long periods of time or intracellular storage pools continuously replenish FVIII-MHC class II complexes on the plasma membrane. 

### Presentation of FVIII Derived Peptides by Macrophages

**Figure 4:**
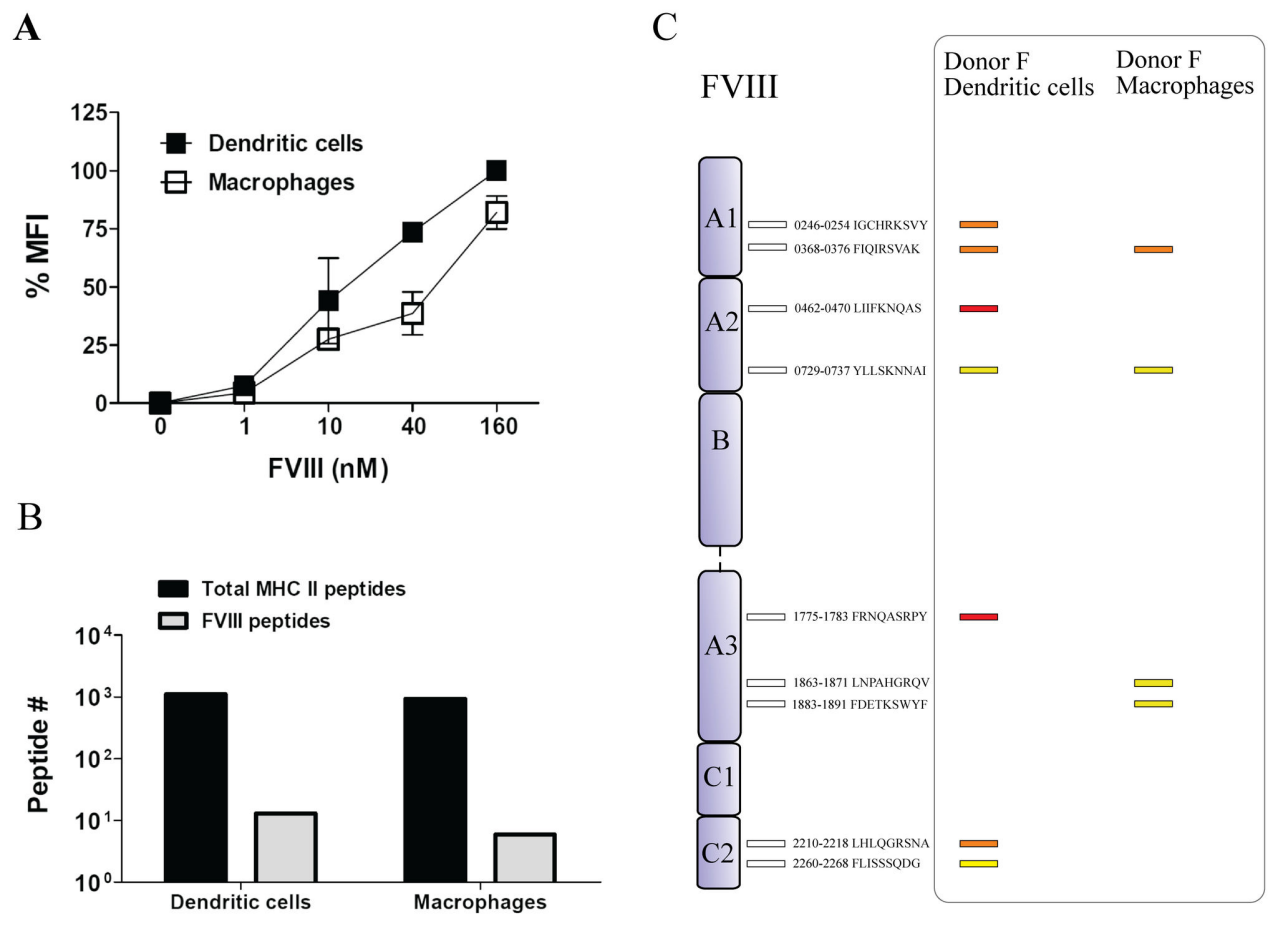
Presentation of FVIII peptides by moDCs and macrophages. **A**. Endocytosis of different concentrations of FVIII by either moDCs or M2 macrophages was measured by flow cytometry. The highest MFI measured in DCs was set to 100%. All other data points are relative to that MFI (expressed as %MFI) B. Cell lysates from FVIII-treated moDCs or macrophages were used to purify HLA-DRB1-presented peptides using an anti-MHC class II antibody. Graph shows quantification of individual MHC class II-bound FVIII peptides. **C**. Schematic representation of FVIII core peptides presented by dendritic cells and macrophages generated from monocytes of donor F.

In order to be able to directly compare the antigen-presenting capacity of different cell types, monocytes from healthy donors were differentiated in parallel into either DCs or macrophages, as described in the Materials and Methods. As reported previously, both macrophages and DCs are able to efficiently internalize FVIII (Figure 4A) [10,14,15]. It has been suggested that *in vivo* macrophages, are primarily involved in endocytosis of FVIII [10]. Therefore, we investigated whether human macrophages are capable of presenting FVIII on MHC class II to a similar extent as human moDCs. The total number of MHC class II-presented peptides recovered from macrophages and dendritic cells was similar (Figure S2A in File S1). However, less FVIII peptides were recovered from FVIII-pulsed macrophages when compared to moDCs (Figure 4B and 4C, Figure S2 in File S1). Macrophages do not only present less FVIII peptides, but also a different and smaller repertoire of FVIII peptides when compared to DCs. Moreover, analysis of the average intensities of FVIII peptides indicates that the majority of them is presented more efficiently by DCs when compared to macrophages (Figure S2 in File S1). 

### Ovalbumin derived peptides are efficiently presented on MHC class II by human monocyte-derived DCs (moDCs)

**Figure 5:**
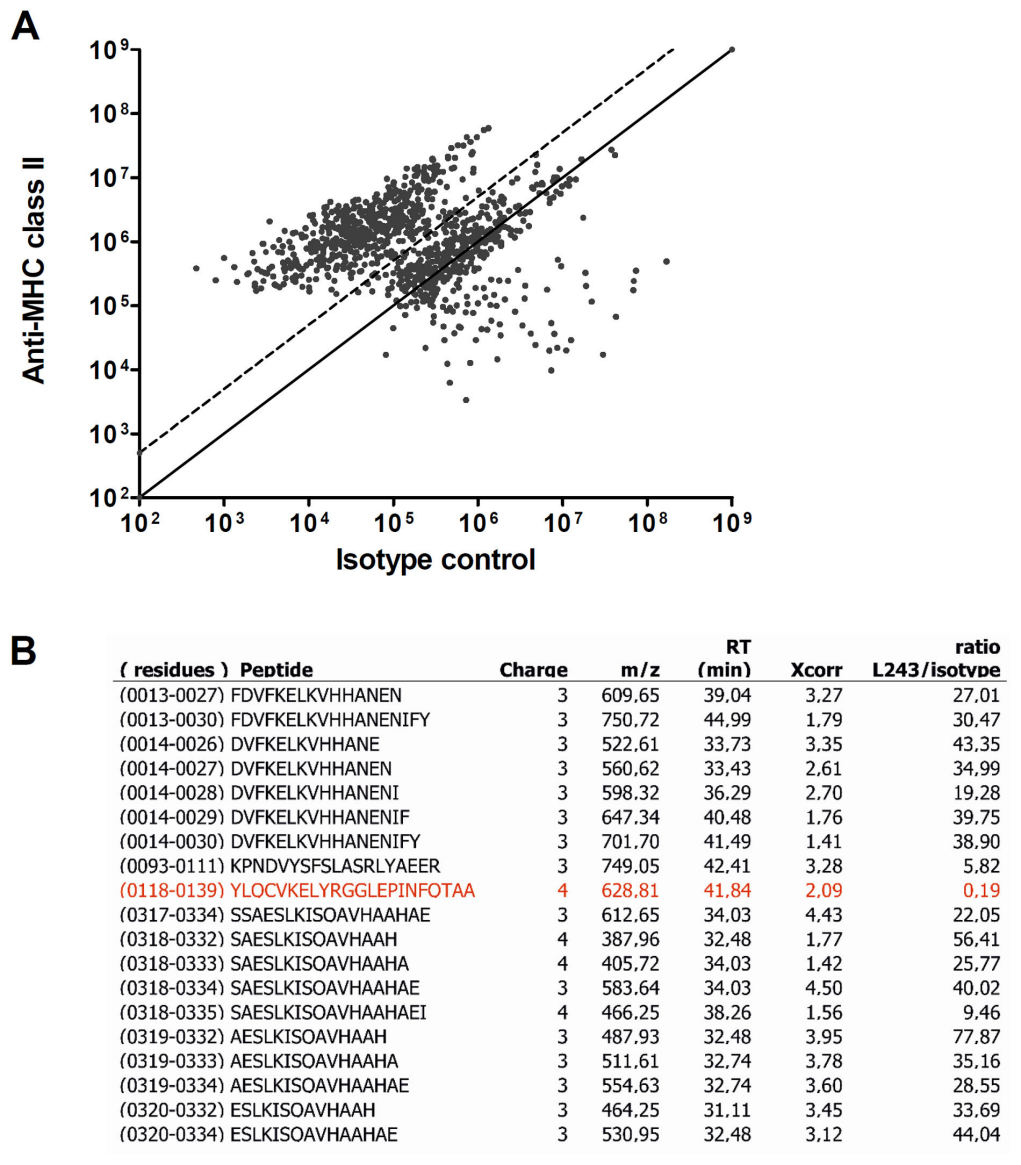
Presentation of OVA peptides by moDCs. **A**. Ovalbumin-derived peptides were identified by mass spectrometry after purification of HLA-DRB1 from lysates of ovalbumin-pulsed cells with either an anti-HLA-DR antibody or an isotype antibody. SIEVE was used to compare intensities of individual peptides and average intensities of each identified peptide are plotted. The diagonal line indicates an equal intensity under each conditions and the dotted lines indicate 5-fold differences in intensity. Peptide clusters with a fold change of 5 or higher were considered as true MHC class II-bound peptides. Only peptides that were sequenced by MS/MS are depicted. **B**. Ovalbumin peptides identified in this experiment are listed with sequence, charge, mass/charge ratio (m/z), retention time (RT), Xcorr value and HLA-DR/isotype ratio. This is the ratio of intensity with which the peptide was detected in the anti-MHC class II pulldown (L243) over the intensity with which it was detected in the pulldown with the isotype control antibody. Peptides excluded due to a ratio lower than 5 are marked in red.

In the previous paragraphs we have documented the cellular requirements for presentation of FVIII derived peptides on MHC class II. To further establish the potential relevance of our experimental system for other antigens we explored whether pulsing of moDCs with a different commonly used soluble antigen – ovalbumin (OVA) resulted in presentation of ovalbumin-derived peptides on moDCs (Figure 5A and B). OVA-pulsed DCS were lysed and cell lysates were immunoprecipitated using either an anti-MHC class II antibody or an isotype control antibody (Figure 5A). Only peptides with a five-fold or higher relative abundance in the MHC class II pulldown are considered true MHC class II-presented peptides. 19 peptides, representing trimmed variants of 3 core peptide sequences derived from OVA, were identified (Figure 5B). Only one OVA peptide was considered not a true MHC class II-presented peptide, indicated in red (Figure 5B). Part of the peptides identified covered the well-documented 323-339 region comprising the epitope recognized by murine ovalbumin specific OT-II cells [17,18]. Collectively, these results show that similarly to FVIII, moDCs can present peptides derived of multiple regions of the widely used model antigen ovalbumin. 

### Repertoire of FVIII peptides presented on MHC class II molecules

**Figure 6:**
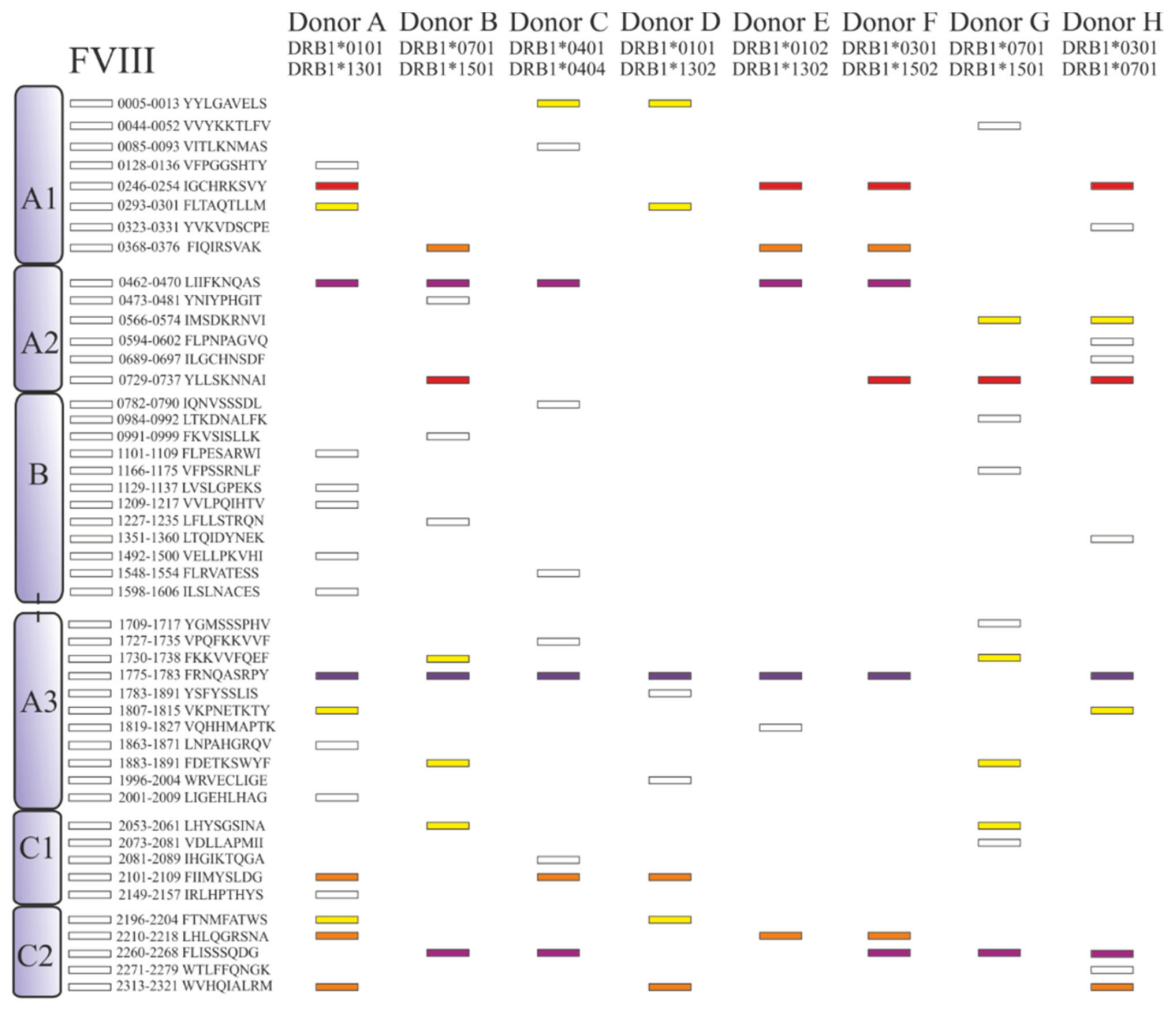
Distribution of FVIII core peptides in eight different donors. Different core peptides are presented by different donors. FVIII-derived HLA-DRB1-presented peptides are represented as rectangles for each individual donor. Depicted in color coding are sequences that are common between two or more donors. Yellow: 2 donors, orange: 3 donors, red: 4 donors, violet: 5 donors and purple: 7 donors. The different FVIII domains and the amino acid sequence of the peptides are depicted schematically.

**Figure 7:**
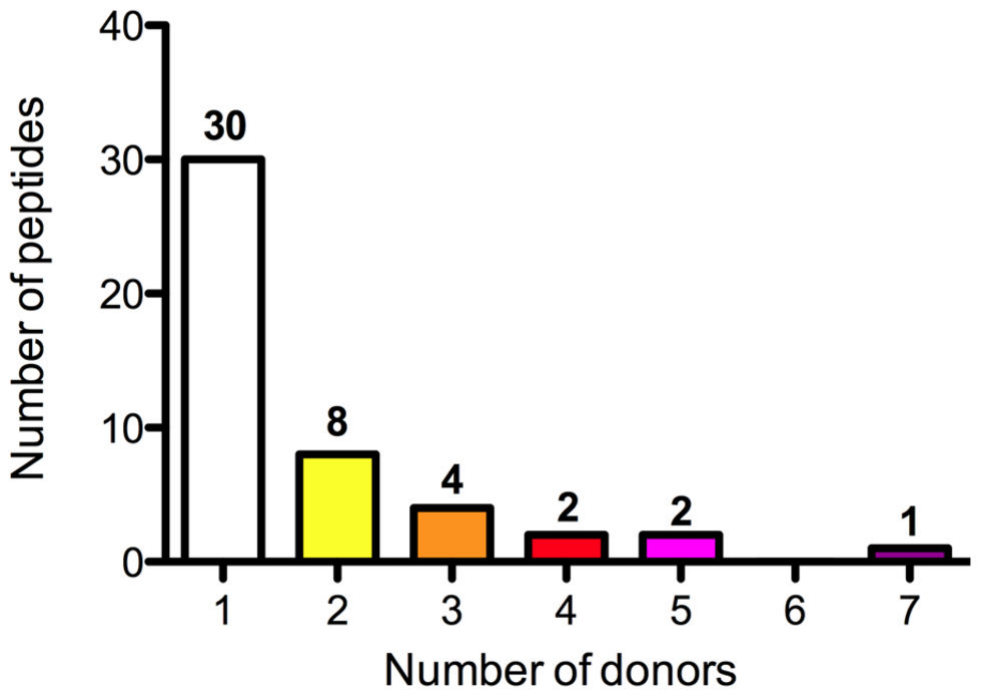
Promiscuity plot of FVIII peptides presented on HLA-DRB1. The number of peptides presented in single or multiple (2-7) donors is indicated. The number of peptides is plotted on the y-axis. The number of donors positive for an individual peptide is displayed on the x-axis.

Previously, we have reported that moDCs derived from 4 different donors with diverse HLA-DR haplotypes present different regions of FVIII [8]. Here we extended these findings by analyzing FVIII peptides repertoire presented by moDCs derived from 4 additional donors. In a similar fashion as described for ovalbumin (Figure 5A), isotype-control immunoprecipitations were routinely performed on FVIII-pulsed moDCs to distinguish between true HLA-DR-binding peptides and aspecifically precipitated peptides. As described previously [8], such control experiments resulted in the identification of FVIII peptides with a length exceeding 25 amino acids, all of them containing an identical core sequence. Such peptides were excluded from subsequent analysis. The results from all donors studied are assembled in Figure 6; core-peptide sequences derived from the FVIII peptides identified for all 8 donors are depicted. Three promiscuous peptides are presented by moDCs derived from at least 5 out of 8 donors (Figure 7). Core-peptide sequence FRNQASRPY (1775-1783, A3 domain) is presented by 7 out of 8 donors. A2 domain-derived core-peptide sequence LIIFKNQAS (0462-0470) and C2 domain-derived core-peptide FLISSSQDG (2260-2268) are presented by 5 out of 8 donors. Two peptides are presented by 4 different donors, 4 peptides by 3 different donors and 8 core-peptide sequences are presented by 2 donors only (Figure 7). The vast majority of identified peptides (30) is presented by moDCs derived from a single donor. These data show that only a limited number of FVIII peptides is presented by multiple HLA-DR molecules. The HLA-DRB1 haplotype of donor B and G is identical (HLA-DRB1*0701/*1501); nevertheless only 5 out of 10 (donor G) or 11 (donor B) peptides are presented by both donors. Similarly, there are only 5 overlapping regions between donor A and D, which are highly similar in DRB1 haplotype. These findings indicate that the repertoire of FVIII peptides presented on MHC class II is not exclusively shaped by polymorphisms within HLA-DR. 

## Discussion

In this study we show that a previously established method for peptide presentation on HLA-DR can be used to study the contribution of various cellular processes to the repertoire of antigen-specific peptides that is presented on MHC class II. Our results indicate that in vitro generated antigen-presenting cell types can be used to study the physiological aspects of presentation of model antigens such as FVIII and ovalbumin on MHC class II. In total, 47 different FVIII core-peptides have been identified in this and our previous study [8]. It is important to note that T-cell reactivity towards the majority of these peptides has been shown in a clinical or experimental setting. For six out of all FVIII regions identified in this study and our previous work [8], reactive CD4^+^ T cells have been identified either in a hemophilia A patients or hemophilia mice [11,19-23]. In addition, another six peptides described in this paper were recently identified as epitopes in a novel human HLA-DRB1*1501 transgenic mouse model for hemophilia A [24,25]. Comparison of peptide-repertoires presented by donors that share identical or closely related HLA-DRB1 alleles suggests only a limited overlap. Although the limited number of donors makes it difficult to draw strong conclusions regarding HLA-restrictions of peptide presentation, our results suggest that additional donor-specific factors could also modulate the repertoire of presented FVIII peptides. Degradation of internalized antigens for presentation on MHC class II is dependent on the proteolytic activity of a variety of proteases such as cathepsins B, E, G, L, S and asparagine-specific endopeptidase (AEP) [26-30]. The expression and/or activity of these enzymes and the expression of FVIII-internalizing receptors can differ between donors. Both monocyte-derived DCs and macrophages readily internalize FVIII, but DCs are notably more efficient in presentation of FVIII peptides on HLA-DR. In our study, macrophages only presented a limited number of FVIII peptides, which were different than those presented by DCs derived from the same donor. This observation further underscores that cellular context contributes to the efficiency and diversity of FVIII peptides presented on MHC class II. 

In this study we show that immature moDCs are able to present antigenic peptides on MHC class II, albeit less efficiently than LPS-matured moDCs. Most FVIII peptides loaded on HLA-DR in mature moDCs are presented on the plasma membrane. In contrast, HLA-DR molecules loaded with FVIII peptides in immature moDCs are residing both inside the cell and on the plasma membrane. This indicated that immature DCs are less efficient in presenting FVIII-derived peptides to CD4^+^ T cells, however they are not completely incapable of doing so. Immature moDCs express only low levels of co-stimulatory molecules such as CD80, CD83 and CD86, which makes them suitable inducers of immune tolerance towards FVIII. Such concept was already verified in previous studies, where treatment of hemophilic mice with FVIII-pulsed immature DCs was shown to induce the expansion of regulatory T cells and inhibit FVIII-specific immune responses [31,32]. Presentation of FVIII peptides on HLA-DR peaks between 12 and 24 hours after maturation and levels off to about half the amount of peptides when maturation takes place for more than 48 hours. DCs are most likely able to retain their capacity of FVIII presentation because maturation induces a decrease in turnover of MHC class II molecules [33,34]. Another explanation could be that FVIII peptides are continuously replenished from intracellular depots as previously shown for ovalbumin [35]. Immature DCs are also able to present antigenic peptides on MHC class II, but the turnover of these complexes is much higher, making immature DCs less effective in presentation of antigens to CD4^+^ T cells [36]. 

Previously, we have shown that the repertoire of FVIII peptides presented on the surface of APCs is strongly influenced by the HLA-DRB1 allele of the donor [8]. Our current data provide additional evidence for presentation of a limited number of FVIII peptides that can bind to multiple HLA-DRB1 molecules. The majority of FVIII-derived peptides identified in this study is presented by moDCs derived from a single donor (Figure 4). Only 8 peptides are presented by moDCs derived from 3 or more different donors (Figure 4). This suggests that each individual presents a set of “private” FVIII peptides. The mechanisms underlying the diversity of HLA-DRB1 presented peptides are currently unclear. The differences observed between DCs and macrophages indicate that factors that regulate the proteolytic activity within APC may also shape the repertoire of HLA-DRB1-presented FVIII peptides. Several cytokines, such as IL-10, IFNγ and TNFα, are able to induce changes in the presentation of antigens by changing the expression or activity of intracellular proteases [28,30,37-39]. Because some of these cytokines are known to have impact on the risk of inhibitor formation in hemophilia A patients, it would be interesting to study effects of these cytokines on the presentation of FVIII on MHC class II. It should be noted that the clinical relevance of our findings is crucially dependent on the specificity and hierarchy of FVIII-specific CD4^+^ T cells present in hemophilia A patients, of which only limited data is available. Modification of putative CD4^+^ T-cell epitopes in the FVIII molecule has been proposed to result in an HLA-dependent reduction of FVIII immunogenicity [19,40]. The current findings may therefore assist in the search for FVIII-specific CD4^+^ T cells in hemophilia A patients and could thereby contribute to the development of FVIII variants with reduced immunogenicity. 

## Supporting Information

File S1 **Figure S1. Subcellular fractionation of immature and mature moDCs on a sucrose density-gradient.** Presentation of FVIII was compared between immature DCs and LPS-matured DCs. Cells were homogenized and PNS was fractionated on a sucrose density-gradient. A. Fractions from the sucrose gradient were analyzed by ELISA for MHC class II molecules and divided into 3 pools per sample as indicated. B. Peptides from each pool were identified by mass spectrometry after tryptic digestion. Identified proteins were annotated based on subcellular localization or function using the Database for Annotation, Visualization, and Integrated Discovery (DAVID) Bioinformatics Resource. C. Accession numbers of proteins identified in the different subcellular fractions. **Figure S2, Presentation of FVIII peptides by moDCs and macrophages.** A. Intensity plot showing reproducibly detected peptide ions across duplicate analyses. Total cell lysates from FVIII-treated moDCs or macrophages derived from donor 2 were used to purify HLA-DRB1-presented peptides using an anti-MHC class II antibody. SIEVE was used to compare intensities of individual peptides and average intensities of each identified peptide are plotted. The diagonal line indicates an equal intensity under each condition and the dotted lines indicate 2-fold differences in intensity. B. FVIII peptides identified in this experiment are listed with sequence, average intensity in cell lysate from macrophages, dendritic cells and intensity ratios between those two conditions.
(PDF)
