# Supplementary material for: Limited Promiscuity of HLA-DRB1 Presented Peptides Derived of Blood Coagulation Factor VIII
Source: PLoS One. 2013 Nov 14;8(11):e80239. doi: 10.1371/journal.pone.0080239 (PMC3828219; doi:10.1371/journal.pone.0080239)
Supplement: File S1 — Figure S1. Subcellular fractionation of immature and mature moDCs on a sucrose density-gradient. Presentation of FVIII was compared between immature DCs and LPS-matured DCs. Cells were homogenized and PNS was fractionated on a sucrose density-gradient. A. Fractions from the sucrose gradient were analyzed by ELISA for MHC class II molecules and divided into 3 pools per sample as indicated. B. Peptides from each pool were identified by mass spectrometry after tryptic digestion. Identified proteins were annotated based on subcellular localization or function using the Database for Annotation, Visualization, and Integrated Discovery (DAVID) Bioinformatics Resource. C. Accession numbers of proteins identified in the different subcellular fractions. Figure S2, Presentation of FVIII peptides by moDCs and macrophages. A. Intensity plot showing reproducibly detected peptide ions across duplicate analyses. Total cell lysates from FVIII-treated moDCs or macrophages derived from donor 2 were used to purify HLA-DRB1-presented peptides using an anti-MHC class II antibody. SIEVE was used to compare intensities of individual peptides and average intensities of each identified peptide are plotted. The diagonal line indicates an equal intensity under each condition and the dotted lines indicate 2-fold differences in intensity. B. FVIII peptides identified in this experiment are listed with sequence, average intensity in cell lysate from macrophages, dendritic cells and intensity ratios between those two conditions. (PDF) [file pone.0080239.s001.pdf]

Combined Supporting Information:

"Limited promiscuity of HLA-DRB1 presented peptides derived of blood coagulation factor VIII"
van Haren *et al*.

Supplemental Figure S1.

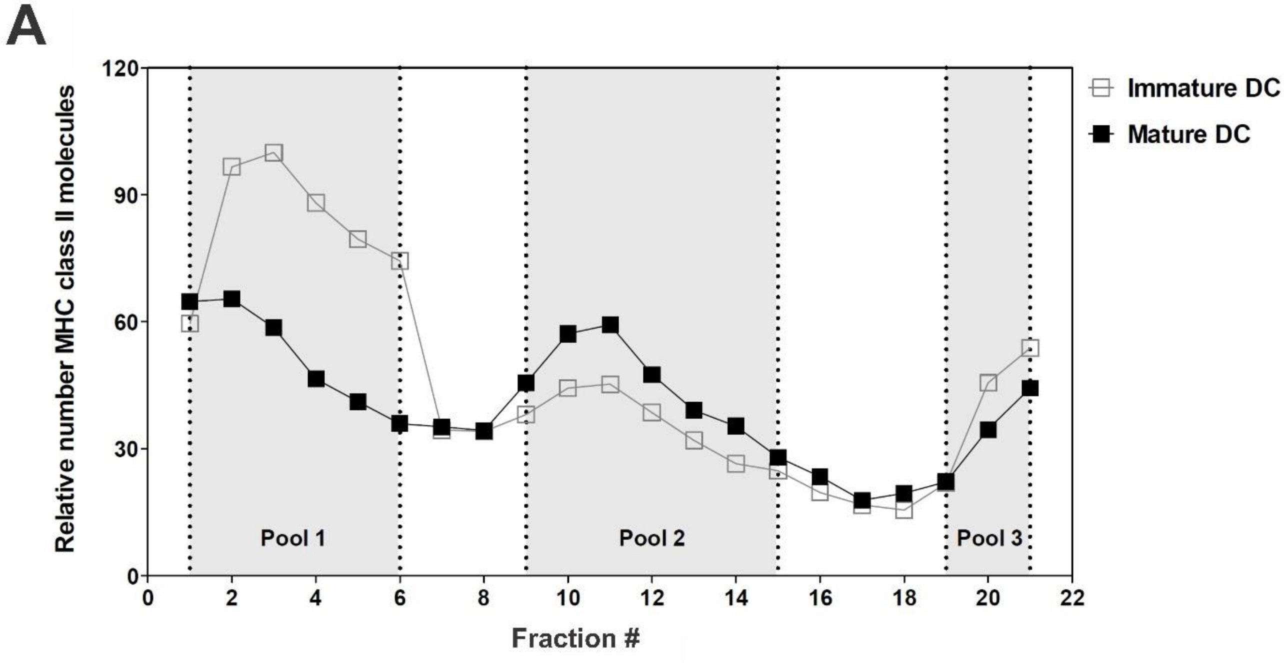

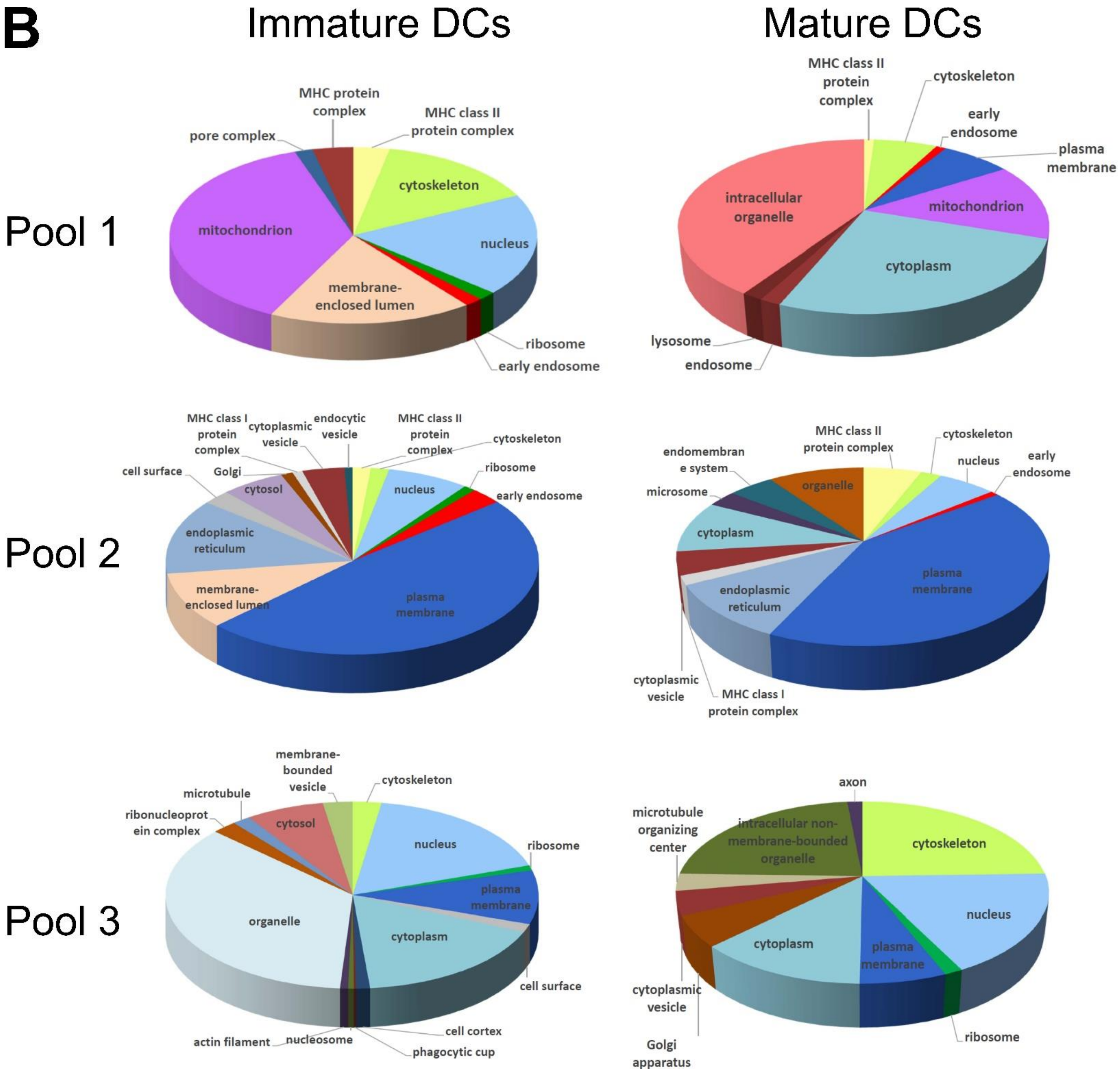
B
Immature DCs
Mature DCs
Pool 1
Pool 2
Pool 3
MHC protein complex
pore complex
MHC class II protein complex
cytoskeleton
mitochondrion
nucleus
membrane-enclosed lumen
ribosome
early endosome
MHC class II protein complex
cytoskeleton
early endosome
plasma membrane
intracellular organelle
mitochondrion
cytoplasm
lysosome
endosome
MHC class I protein complex
cytoplasmic vesicle
endocytic vesicle
MHC class II protein complex
cytoskeleton
cell surface
Golgi
cytosol
nucleus
ribosome
early endosome
endoplasmic reticulum
membrane-enclosed lumen
plasma membrane
endomembrane system
MHC class II protein complex
cytoskeleton
nucleus
early endosome
microsome
organelle
cytoplasm
endoplasmic reticulum
plasma membrane
cytoplasmic vesicle
MHC class I protein complex
membrane-bounded vesicle
microtubule
cytoskeleton
ribonucleoprotein complex
cytosol
nucleus
ribosome
plasma membrane
organelle
cytoplasm
cell surface
actin filament
nucleosome
cell cortex
phagocytic cup
axon
microtubule organizing center
intracellular non-membrane-bounded organelle
cytoskeleton
nucleus
cytoplasm
plasma membrane
cytoplasmic vesicle
Golgi apparatus
ribosome

C

Immature DCs Pool 1	UniProtKB entry
MHC class II protein complex	Q13365, Q7YPT8, P01913, Q30178, Q7YPT7, P79483, P20039, Q29814, P04229, P01919, B4E328
cytoskeleton	P46940, P04259, P35527, Q9Y4G6, Q9Y5K6, Q6ZUZ3, Q99456, P13645, P58107, P13647, P08670, P04264, Q7Z4R3, Q5XUU0, P78559, P19012, Q86VQ2, P08779, A2A3Q7, Q0IIN1, Q5T0H7, Q562R1, Q99456, P13645, P19012, P08670, P13647, P35527, P35908, Q0IIN1O43896, Q07283, Q13707, P35908, Q9BXW4, Q9GZY0
nucleus	P46940, Q7Z5Y1, Q6VMB5, B7Z7S9, Q9Y5K6, Q9BTT4, O15047, P12270, Q53T03, Q9NXY2, P35232, P49792, Q8TEQ6, B1A8Z4, Q14397, P62805, P06702, Q96I25, P62988, Q9H089, Q5T7C6, P10809, Q9H2P0, O43143, P26583, Q9GZY0
ribosome	P62988
early endosome	P61769, Q9Y2I7, P10809, P30048
membrane-enclosed lumen	P46940, Q5BJH1, P00367, Q6VMB5, Q7Z5Y1, Q1L6K6, P30405, Q5QNZ2, Q9Y5K6, O15047, Q9BTT4, P21796, P05141, P12270, P61604, Q9NXY2, P35232, Q8TEQ6, Q9UDG6, Q14397, P62805, P06702, P40926, P14854, P49411, P00451, P38646, B4DIH1, P62988, P06576, Q04837, Q5T7C6, Q9H089, P10809, Q9H2P0, P26583, P30084, P55084, P40939, P25705, Q99798, Q9GZY0
mitochondrion	P00367, P48735, P30405, O00483, Q5QNZ2, P21796, P61604, P18859, P22695, Q9UDG6, Q13423, P40926, P49411, Q9Y6N5, Q13011, O75323, P56385, P38646, Q5JXL8, P13073, Q04837, P10606, P10809, Q5BJH1, Q1L6K6, P09669, Q9BWM7, P61221, P05141, P35232, P30048, P14854, P14927, Q567R0, P12236, P20674, P07203, O75879, Q6IB91, B4DIH1, P06576, P31930, P48047, P30084, P40939, P55084, P25705, P21397, Q99798
pore complex	P12270, Q53T03, P49792, P21796
MHC protein complex	P61769, Q13365, Q7YPT8, P01913, Q30178, Q7YPT7, P79483, P20039, Q29814, P04229, P01919, B4E328

Immature DCs Pool 2	UniProtKB entry
MHC class II protein complex	Q30178, P79483, P20039, P04229, Q30155, P01919, B4E328, P01911, P01903, Q13365, P01913, Q7YPT8, Q7YPT7, Q29814
cytoskeleton	P46940, P19105, P20929, A6NIV8, P15311, Q9HD67, Q9Y5K6, Q9Y4G6, Q9Y623
nucleus	P10412, P04908, A8MQC5, Q02539, P16402, Q96A08, P16401, Q16777, P62805
ribosome	P60866, P62988, Q6DKI1
early endosome	P61769, Q5SPM3, P61106, Q9UL26, Q9BV40, Q3HNF7, P04439, P01891, Q59GU6
plasma membrane	P46940, P13987, P20039, P01893, P01919, P09601, B0FYA8, Q95604, Q5VWH6, Q306H8, P01911, O14514, P22897, P61769, P61106, P01913, Q9UL62, O75695, P17661, Q9UDG2, Q86YS6, B4DF05, Q9Y490, P00451, P04229, Q969V1, Q6UVK1, P20645, P01903, Q13349, Q29814, Q3HNF7, P07237, P01891, P98172, P16150, Q7Z669, P61026, Q30155, Q9UQ52, Q9Y4G6, B4E328, B7Z3U6, P35232, P84095, P07942, P11215, P61225, P19087, P08754, P43005, Q7YPT8, P06576, Q7YPT7, Q8TCR4, P63218, Q86XU3, Q4W6C4, O15085, Q9HDC5, Q30178, Q14573, Q8WYU9, Q9UKZ4, Q6N096, P18084, Q96JW4, Q59H50, Q5SPM3, P15311, P51572, B7Z1E7, P04264, Q53EY4, Q9UL26, P04439, Q59GU6, P17693, P04839, P00742, Q8N537, P20702, P27797, P17931, P14207, Q9Y5K6, Q7RTM4, P56373, Q13365, P62873, Q9BRL5, B2RTS2, P08670, Q6FIG4, Q9P0L0, Q6ZMQ2, P79483, Q92953, Q86UE4, Q14517, Q04941, P51148, Q9BX59, P15144, Q10588
membrane-enclosed lumen	P46940, Q14573, P62072, P09601, Q2KHP4, Q59GH7, P05455, P62826, Q9UDG2, Q9UQE7, Q5VUQ9, Q9Y5N6, P06702, Q9H6E5, B4DUW6, P00451, Q96DD3, O60244, Q99638, O95881, Q9BVC6, P07237, Q5T6W5, Q9GZY0, Q15397, Q9BS26, P12755, Q9UIJ7, Q9H2Y7, P27797,

	Q9Y5K6, O15047, Q9BTT4, P13667, P35232, P14625, Q9BRL5, Q8NEM1, Q96AG4, Q14397, P62805, P30101, P35558, Q86UE4, P30040, Q9ULW3, P62988, P06576, Q5T7C6, Q9H089, P23284, P15144, Q9NYH9, O15085
**endoplasmic reticulum**	Q16696, Q14573, Q9HDC5, Q12907, Q9Y6K0, P39656, P27797, P09601, Q14165, Q2KHP4, P16435, P13667, Q96SP2, P11509, P61106, P14625, Q9H3N1, Q96AG4, Q9BS14, P51572, P04843, Q9UDG2, P51571, Q9P0L0, P30101, B4DF05, Q86UE4, A8K318, P30040, Q04941, P01903, Q9H089, Q15392, P23284, O95881, Q9BVC6, P07237, Q8N394, Q9BX59, Q15005, Q15397, Q9BS26
**cell surface**	P16150, P13987, P27797, Q6UVK1, Q2KHP4, P20645, P01903, P00742, P06576, P20702, P07237, Q9UDG2, P11215
**cytosol**	P17812, Q9H2Y7, Q6ZU43, P27797, P09601, Q2KHP4, P61106, P15311, P14625, Q16644, Q9BRL5, P08670, A8MXQ4, P51572, Q9BYX7, P49888, P62826, Q9UDG2, Q5T6R6, Q14397, P60866, Q6QE17, P68104, Q9Y490, P35558, P07196, P62988, O00459, P15144, O15085
**Golgi**	Q12907, P49755, P07237, P15144, Q2KHP4, Q9BS26
**MHC class I protein complex**	P61769, P17693, Q5SPM3, P01893, Q3HNF7, P04439, P01891, Q95604, Q306H8, Q4W6C4
**cytoplasmic vesicle**	P08754, P30101, Q6ZMQ2, P49755, P00451, P30040, P51149, Q9Y5K6, Q2KHP4, B7Z3U6, P51148, P13667, P61106, Q8TCR4, P14625, P23284, Q9H1R3, P07237, Q9BS14, P04843, P62826
**endocytic vesicle**	Q8TCR4, Q9Y5K6, P51149, P51148

Immature DCs Pool 3	UniProtKB entry
**cytoskeleton**	P07437, Q7LE35, Q6AWB3, Q17RI8, Q6PEY2, P68371, O43264, P33176, P15311, Q9BRL5, Q7Z3Z5, B1ALC3, Q86VS8, Q9NQT8, Q13509, Q9P1R8, Q969V4, Q13464, P68363, Q658K6, Q9BYG9, Q76KD6, O95613, P04792, Q96DT5
**nucleus**	P46940, Q9UPN6, P52597, Q9Y6Q2, Q9C0E2, B7ZLQ3, P14651, Q9NYB0, B7Z7S9, P23528, Q17RI8, Q5H924, Q2KHP4, P23527, P07737, Q53T03, P49792, Q6IQ32, O95071, O43707, Q8IXH6, Q99497, Q5HY54, Q5VUQ9, Q05639, P51826, P04080, P06702, P59047, Q9H6R9, P04083, Q9H9Y2, P06703, B2RU05, O75368, Q06HB5, Q5JXT4, Q13344, O00299, P42704, B0JZA6, P14317, Q8IY18, P61586, Q9NU22, P15259, O14646, Q99536, Q14974, Q96K58, Q9P127, P49005, Q16650, P35609, Q2NL82, O43264, Q6P1J9, Q6ZP55, B4DRN2, Q8WUY8, Q59FF6, Q15906, P55072, P20393, P63104, P10073, Q59HE6, Q9Y473, Q92618, A8K4S5, P35716, Q8IY81, Q9H0E7, Q6PKH8, Q5RI19, P62988, Q9Y2U8, P40425, Q4VY20, Q9UL17, Q09161, Q9UEW8, B1AJP8, Q7LE35, Q96KK5, Q5T8A7, O75312, P60709, B3KX84, Q9HBT7, Q9NQ03, A7E2F8, Q3MHU9, P31949, Q9BQ02, Q5TCW7, Q06830, O95411, P61011, P33778, P78371, P31629, Q6QHK4, O75467, Q15022, Q07954, Q9BYG9, P26447, Q9HCD5, Q8IVT8, P78367, Q5T6W8, P50749, P04792, P62191, Q2QD09, Q8NAP3, Q5DTE4, Q59GG2, P46777, P35269, P27797, P17931, Q7RTM4, P15918, Q96C00, O76039, Q02386, P40121, P42285, O95373, P08107, Q9BRL5, P04626, Q7Z3Z5, Q9Y4C8, P09017, P62805, Q14397, B4DNK3, P35453, P51812, Q8IYW5, Q9ULW6, A6NKN2, B3V8S3, P62937, P52945, Q5T7C6, Q86WM5, A0JNZ9, P43243, Q6NXT2
**ribosome**	P62988, P46783, Q9BYG9, P46777, Q9Y3D5, Q6S4P3
**plasma membrane**	B0FYA3, P07437, Q5VTU6, Q30178, Q9UEW8, Q9P244, Q13829, Q5BM92, Q92673, P08133, O14514, P15311, Q8TCU5, O15554, A6NFI4, P27037, P31146, Q9UPW8, P04083, Q5TBN5, O43520, Q9Y490, Q86SW9, Q4G0X4, P04229, Q13797, Q59GU6, Q6TV07, P22413, Q07954, P54764, P00742, Q92839, P61586, P14550, P61158, P13796, Q00013, P29317, Q7LBE3, Q8N4C1, Q9UJ43, P52565, O00559, O95049, P27797, Q5JR08, Q8N1C3, Q6SV52, P15328, Q9Y4G6, Q7RTM4, P35609, Q9BXV5, O43868, B4DRN2, P04626, P13725, Q15739, A5JJ20, A6ND72, Q13509, Q6QEF7, P28222, Q9UEV8, Q5W0I9, Q5JQ19, Q92817, Q5JUA8, B3V8S3, Q0P5W4, Q7YPT8, P16671, A8K7I4
**cell surface**	Q5VTU6, Q9UJ43, P27797, Q5JQ19, O75334, Q6SV52, Q2KHP4, Q5SX88, P22413, Q5RI19, Q07954, P00742, P16671, P04792, A8K7I4
**cytoplasm**	Q5VU64, Q96QK1, P07437, P04406, Q9Y6Q2, P35527, P23528, B2RBL3, Q2KHP4, Q68DF1, P49792, P18859, O43707, P17661, P14649, Q99497, Q5HY54, Q05639, P68104, Q86VS8, P31146, Q9P1R8, P59047, Q5TBN5, Q9Y490, Q9UHN1, P18669, Q13464, Q9Y2K3, Q6S4P3, Q6TV07, Q58FF3, P60842, O00299, P42704, P08238, Q8NFT8, P14317, P10599, P15259,

	P13796, P53634, Q14974, Q9H4K7, Q9BVR0, Q9H2L7, O00559, P52565, P04075, P52566, P00488, P04040, Q9H5Q4, P35609, O43264, Q13094, Q13014, O95817, B4DRN2, P55072, P63104, P30101, P37837, P05787, P21953, O75907, Q6PKH8, Q8WWX9, Q5RI19, Q0P5W4, Q9NRN7, Q658K6, P62988, O43819, Q86U75, O75874, Q8IYI6, Q9NRV9, A8MXA4, Q4VY20, P07355, Q09161, Q8WYU7, P09467, B7ZKZ2, P60709, Q8IWL3, Q6ZWP8, Q7Z2T3, Q5JWR5, Q6PEY2, Q59F84, P68371, Q5SX88, P08133, P15311, Q5JTZ9, Q15034, Q9BQ02, B9ZVW5, Q06830, P40925, P61011, Q9HCS2, P78371, Q06323, Q59GU6, Q07954, Q9BYG9, Q6NT55, Q53FA3, Q9BYN0, P14550, B4DY19, P19113, P61158, O95613, P04792, P62191, Q2QD09, Q6QHC5, Q00013, P20929, Q59GG2, Q5BJH1, P46777, Q6AWB3, P27797, P61981, Q7RTM4, Q16555, Q9UG63, P40121, P33176, Q9BXV5, P08107, Q9BRL5, P08670, Q16753, P04626, Q19KD6, Q96NB2, Q7Z3Z5, Q86SX6, Q14397, A6ND72, B4DNK3, P46783, Q9UHG2, B7Z1Y0, Q6QEF7, Q15751, Q5JQ19, Q5JUA8, Q8IVB4, P15907, B3V8S3, B3KS15, P62937, Q76KD6, Q9Y3D5, P62330, P16671, P07339, Q59GE9, Q504Z1, Q96HY7, Q96EK5, Q9Y623, A8K7I4
**cell cortex**	P31146, P59047, P15311, Q8IYI6, Q9BQ02, P62330, P60709, O43707, P23528, Q00013, A6ND72, Q5HY54
**phagocytic cup**	P31146, Q5TBN5, P13796
**nucleosome**	P33778, Q96KK5, Q6NXT2, P23527, P62805
**actin filament**	P46940, P31146, P15311, Q5TBN5, Q5JUA8, P61158, P13796, P35609
**organelle**	P46940, Q9C0E2, P14651, P23528, Q5H924, P23527, P07737, Q68DF1, P49792, Q8IXH6, Q5HY54, Q5VUQ9, Q86VS8, P59047, Q9UHN1, Q9H9Y2, Q13464, O75368, Q6TV07, Q58FF3, Q5JXT4, Q13344, Q8NFT8, P14317, P61586, P53634, Q99536, Q9H4K7, Q9H2L7, Q96K58, P52566, Q9P127, P00488, P04040, P35609, O43264, Q6ZP55, Q8WUY8, P55072, P63104, P30101, P10073, A8K4S5, P35716, Q8IY81, P21953, Q6PKH8, Q8WWX9, P62988, O75874, Q9NRV9, Q9UL17, Q8WYU7, Q09161, Q9UEW8, Q7LE35, Q5T8A7, Q96KK5, B7ZKZ2, O75312, P60709, Q8IWL3, Q6ZWP8, Q7Z2T3, Q59F84, Q9HBT7, P08133, Q9NQ03, Q5JTZ9, Q15034, P31949, Q06830, O95411, P33778, Q9HCS2, P31629, Q6QHK4, O75467, Q15022, Q07954, Q9HCD5, Q6NT55, Q5T6W8, P04792, Q8NAP3, Q5BJH1, P46777, P27797, Q7RTM4, O76039, Q02386, P33176, P40121, P42285, P08107, Q9BRL5, Q19KD6, Q7Z3Z5, Q9Y4C8, Q86SX6, Q14397, P51812, Q8IYW5, Q15751, B3V8S3, P52945, Q9Y3D5, P62330, P43243, Q6NXT2, Q96HY7, Q96EK5, Q96QK1, Q9UPN6, P52597, Q9Y6Q2, B7ZLQ3, B7Z7S9, Q9NYB0, Q17RI8, Q2KHP4, Q53T03, Q6IQ32, O95071, P18859, O43707, Q99497, P51826, Q05639, P04080, P06702, P31146, P04083, Q9H6R9, Q9Y490, P06703, B2RU05, Q06HB5, O00299, P42704, P08238, B0JZA6, Q8IY18, P10599, Q9NU22, P15259, O14646, Q14974, Q9BVR0, O00559, P04075, P49005, Q9H5Q4, Q16650, Q2NL82, Q6P1J9, Q13014, B4DRN2, Q59FF6, Q15906, P20393, Q59HE6, Q9Y473, Q92618, Q9H0E7, O75907, Q5RI19, Q0P5W4, O43819, Q9Y2U8, Q86U75, P40425, A8MXA4, Q96DT5, Q4VY20, P07355, B1AJP8, Q5JWR5, B3KX84, Q5SX88, A7E2F8, Q3MHU9, Q9BQ02, Q5TCW7, P61011, P78371, Q59GU6, Q9BYG9, P26447, Q8IVT8, P78367, B4DY19, P50749, P61158, P62191, Q6QHC5, Q2QD09, Q5DTE4, Q59GG2, P35269, P17931, Q96C00, P15918, Q9UG63, Q16555, O95373, Q9BXV5, Q16753, P04626, Q96NB2, P09017, P62805, B4DNK3, Q9UHG2, P35453, B7Z1Y0, Q9ULW6, Q5JUA8, Q8IVB4, A6NKN2, P15907, P62937, Q5T7C6, Q86WM5, A0JNZ9, P07339, P16671, Q59GE9, A8K7I4
**ribonucleoprotein complex**	Q09161, P46783, P52597, Q59HE6, P46777, P61011, P27797, P60709, Q6S4P3, Q5RI19, P62988, Q5JXT4, P42285, Q9BYG9, P08107, Q9Y3D5, Q9BQ02, Q59GE9, O43707
**microtubule**	Q86VS8, Q9NQT8, P07437, Q13509, Q7LE35, Q969V4, Q6AWB3, P68363, Q6PEY2, Q17RI8, P68371, O43264, P33176, Q9BRL5, B1ALC3, Q96DT5
**cytosol**	Q96QK1, Q09161, P07437, P09467, P60709, B2RBL3, P68371, Q2KHP4, P15311, P49792, B9ZVW5, Q99497, Q5HY54, P68104, Q9P1R8, P40925, P59047, Q5TBN5, Q9Y490, P18669, P78371, Q06323, Q13464, Q6S4P3, P60842, Q9BYG9, O00299, P10599, Q9BYN0, P14550, P15259, P19113, O95613, P62191, P13796, Q2QD09, Q59GG2, Q14974, P46777, P52565, P27797, Q6AWB3, P04040, P61981, Q13094, Q13014, Q9BRL5, O95817, B4DRN2, P08670, Q19KD6, P55072, A6ND72, Q14397, B4DNK3, P46783, P37837, Q6QEF7, Q15751, O75907, B3KS15, Q9NRN7, P62988, P62937, O75874, Q9NRV9, Q4VY20
**membrane-bounded vesicle**	P07355, B7ZKZ2, P52566, P04075, P00488, P35609, Q2KHP4, P08133, P40121, Q15034, O43707, Q06830, P63104, B4DNK3, P31146, P30101, Q9UHG2, Q6TV07, B3V8S3, Q07954, P08238, P16671, P07339, B4DY19, Q4VY20, A8K7I4

Mature DCs Pool 1	UniProtKB entry
**MHC class II protein complex**	Q307W5, Q30178, P79483, P20039, P04229, P01919, P01911, Q13365, P01913, Q7YPT8, Q7YPT7, Q29814, Q29967
**cytoskeleton**	P20929, Q9ULI4, P04259, Q04695, P36404, P35527, P60709, Q9UIF3, Q9Y5K6, Q6ZUZ3, Q16352, O95678, P07737, P13645, B9ZVU5, P13647, P08670, P02533, P04264, Q5VU72,

	P04083, B4DHI8, P19012, Q92817, P08779, Q6N086, P49454, Q9BW62, Q562R1, P12882, O43896, Q8WU19, Q5SYB0, P35908, Q59FE7, Q9GZY0, Q6ZNL4, P46821
**early endosome**	Q9Y2I7, Q9UL26, P10809, P30048, Q6BDI9, Q96NW4
**plasma membrane**	Q30178, Q5VTU6, P31639, P20039, P01919, P01911, Q02763, P01913, O75695, Q9UL62, A8MTJ3, Q29967, P63096, Q307W5, Q9UPW8, O43520, P04083, P04229, Q13797, O75323, P12882, P49447, P22413, Q8NHU3, P10809, Q29814, Q16099, P27797, B3KWU3, Q9NPC1, Q13365, P05141, P35232, P84095, Q9UM47, Q6ZMQ2, P79483, Q7Z2E7, Q92817, Q14517, Q0P5W4, Q7YPT8, Q7YPT7, Q59GW4, Q5SYB0, P21397, P15144, Q6ZNL4, P46019
**mitochondrion**	P00367, P48735, Q5QNZ2, P21796, P61604, Q6IAL5, P13804, P18859, P22695, Q13423, P40926, Q9Y6N5, P49411, Q13011, O75323, Q9NX63, P38646, P13073, P10606, P10809, Q5VZD9, Q16698, Q9Y6C9, P09669, O95573, P04040, Q96C01, Q9BWM7, P61221, P05141, P35232, P11310, P30048, P00403, Q9BX68, Q5VVL7, P14854, P21912, Q5T851, Q567R0, P12236, P20674, Q9BWL6, P07203, Q9Y2Q3, O75879, P99999, Q6IB91, P06576, P31930, B3KRW1, P48047, P30084, P40939, P55084, P21397, P25705, Q99798, O75390
**cytoplasm**	P04406, P00367, P36404, P35527, A6NK99, P07737, Q59GH7, Q9NXY4, Q6IAL5, P02533, P18859, P22695, Q5VU72, P68104, P06702, P04083, P49411, Q66K63, P00451, Q13011, P20592, Q9BTY4, Q9NZK5, Q562R1, P12882, A8MY65, Q8NHU3, P13073, Q9GZY0, Q5VZD9, P53634, P15586, Q04695, P09669, Q14164, P04040, Q6ZUZ3, P11117, Q96NW4, P61221, Q5QP88, O00754, P11310, P35232, B9ZVU5, Q9UBR2, Q02742, P30048, Q5K651, P09382, P00403, Q9BVW5, Q16842, P08754, P30101, Q5VVL7, B4DHI8, Q92618, P12236, Q92817, O75879, P99999, Q0P5W4, Q6IB91, P06576, P31930, P42338, O43896, Q5SYB0, P55084, P35908, P40939, Q6BDI9, Q59FE7, Q99798, O75390, Q6ZNL4, Q6PD74, P46019, P02794, P60709, P07451, P48735, Q5QNZ2, Q9HBK9, P21796, P61604, A8MTJ3, P13804, Q13423, Q9UPW8, P40926, Q9UL26, Q9Y6N5, O43567, O75323, O60248, Q9NX63, Q9H9H4, P49454, Q6UW63, P38646, P10606, P10809, Q76KP1, O00459, Q8N1K5, P19338, P46821, Q9BPX1, P20929, Q16698, P46777, Q9Y6C9, P27797, O95573, P07711, Q9Y5K6, O15049, Q96C01, Q9BWM7, P05141, Q16548, P08670, A1A4S6, Q9BX68, B4DNK3, Q6ZMQ2, Q9HCU4, A6NIM1, P14854, P21912, Q5T851, Q567R0, P20674, Q9BWL6, Q6N086, P07203, P51149, Q9Y2Q3, Q9Y2I7, B3KRW1, Q59GW4, P48047, Q5UGI3, P30084, P21397, P25705, P15144, Q8N9Q8
**endosome**	Q9Y2I7, Q9NXY4, Q9UL26, P10809, P30048, Q6BDI9, Q9H9H4, P51149, Q96NW4
**lysosome**	O00754, P15586, Q9UBR2, P07711, P04040, P51149, P53634, P11117, Q96NW4
**intracellular organelle**	P00367, Q6VMB5, P36404, P35527, Q14146, P07737, Q59GH7, P13645, Q9NXY4, Q6IAL5, P13647, P02533, P18859, Q1ED39, Q5VU72, P22695, P06702, P04083, P19012, P49411, Q66K63, P00451, Q13011, Q96BN2, O60281, P20592, Q562R1, Q9BTY4, Q9NZK5, P12882, A8MY65, Q8NHU3, P13073, Q9HAN9, Q6MZP9, Q9GZY0, Q5VZD9, P53634, Q04695, Q9ULI4, P15586, P09669, Q9UIF3, P04040, Q6ZUZ3, Q96NW4, P11117, P61221, O15090, O00754, P35232, P11310, Q9UBR2, B9ZVU5, Q02742, P30048, P00403, Q9UM47, Q9BVW5, Q96MF7, Q16842, P08754, P30101, Q5VVL7, B4DHI8, Q92618, P12236, Q92817, Q96NG5, Q504T0, O75879, Q0P5W4, P99999, Q6IB91, P06576, P31930, O43896, Q5SYB0, P55084, P35908, P40939, Q6BDI9, Q9NXD3, Q59FE7, Q99798, O75390, Q6ZNL4, P04259, Q96KK5, P60709, Q15431, P48735, Q5QNZ2, P21796, P61604, A8MQC5, P13804, P04264, Q9H5V7, Q9C002, Q13423, P40926, Q9UL26, Q9Y6N5, Q86VG0, O43567, O75323, O60248, P08779, Q9NX63, Q9H9H4, P49454, Q6UW63, P38646, P49447, P10606, P10809, Q8WTR7, Q56NI9, Q76KP1, Q8N1K5, P19338, P46821, P20929, Q16698, Q5VZB9, P46777, Q9Y6C9, P27797, O95573, P07711, Q9Y5K6, O15049, Q96C01, Q9BWM7, Q16352, O15047, O95678, P05141, P08670, Q9BX68, P62805, B4DNK3, Q6ZMQ2, Q7Z3X9, A6NIM1, P14854, P21912, Q5T851, Q567R0, P20674, Q9BWL6, Q6N086, P07203, P51149, Q9BW62, Q9Y2Q3, Q5T7C6, Q9Y2I7, B3KRW1, Q59GW4, Q8WU19, Q9H2P0, P48047, Q5UGI3, P30084, P21397, P25705, Q8N9Q8

Mature DCs Pool 2	UniProtKB entry
**MHC class II protein complex**	Q30178, P79483, P20039, P04229, P01919, B4E328, Q2PZR0, P01911, Q13365, Q7YPT8, P01913, Q7YPT7, P01907, Q29814
**cytoskeleton**	P19105, P15311, Q16658, Q9Y5K6, Q9Y4G6
**nucleus**	Q92794, Q96KK5, P62805
**early endosome**	P61769, P01891
**plasma membrane**	Q30178, P13987, P20039, P01893, P01919, Q95604, P01911, O14514, P61769, P22897, P48169, P01913, P15311, P51572, Q9UDG2, P04264, Q86YS6, P05362, P04229, Q9HAA0, Q2PZR0, P00742, P01907, Q13349, Q29814, P07237, P01891, P27797, Q59GK8, Q9NPC1, Q9Y4G6, P14207, B4E328, Q9Y5K6, Q13365, P48058, P84095, Q9BRL5, P08670, P11215, P19087, P09848, P79483, Q04941, Q7YPT8, Q7YPT7, Q16658, P15144, P46019, O15085

**endoplasmic reticulum**	P30101, Q9HCS2, P39656, P27797, A8K318, P30040, Q2KHP4, Q04941, P13667, Q9H089, P14625, P23284, P07237, P51572, Q9UDG2
**MHC class I protein complex**	P61769, P01893, P01891, Q95604
**cytoplasmic vesicle**	P13667, P30101, P48058, P14625, P23284, P49755, P07237, P30040, Q9Y5K6, Q2KHP4
**cytoplasm**	P50747, B4DKY2, Q9HAT8, P27797, Q59GK8, Q2KHP4, P62988, P15311, P14625, Q9BRL5, P08670, P51572, Q9BYX7, Q6IQ15, O00459, Q9UDG2, P15144, P49721, O15085, Q14397
**microsome**	P00742, P14625, Q9HCS2, P49755, P39656, P07237, P27797, Q9UDG2
**endomembrane system**	P61769, P48058, P14625, Q9HCS2, P49755, P39656, P51572, P01891, P15144, Q2KHP4, Q04941
**organelle**	Q59HE6, Q92794, Q96EG6, Q96KK5, Q9Y4G6, Q9Y5K6, Q562R1, P62988, P19105, Q59GH7, P15311, P13645, P48058, Q9BRL5, Q16658, P08670, Q9BYX7, P04264, O15085, P62805

Mature DCs Pool 3	UniProtKB entry
**cytoskeleton**	Q5VU64, P46940, P07437, B3KX42, P04259, Q59G59, P35527, P60709, Q9UFG9, P23528, Q5HY93, Q5JVZ5, Q4W4Y1, Q6PEY2, P02730, Q9H7Z5, A8CDT9, P07737, Q14192, P15311, Q8IU65, P35663, P13645, P13647, P02533, Q9UJC3, Q9BYX7, P17661, P04264, Q5HY54, Q9NQT8, P31146, O14757, Q9P1R8, P04083, P19012, Q13464, Q9Y2K3, P68363, P08779, P12883, Q562R1, Q9BYG9, P42704, Q86TH3, P61586, Q9GZY0, O95613, P04792, P13796, A8MW06, Q14019, P52566, P04075, P30086, B7ZMF0, Q9Y4G6, Q6ZPD6, Q16352, Q16555, P40121, A6NIV8, Q9BRL5, P08670, Q5JV91, Q7Z3Z5, Q9UKN7, P63104, Q96P04, P62166, P63313, Q92817, B3V8S3, Q9Y2I6, Q16658, Q07283, Q76KD6, P35908, Q7Z3Y8, Q3MIV8, Q9Y623, Q7Z3Y7
**nucleus**	P12755, Q14573, O60934, P60709, P49005, P35249, Q15699, Q9BRL5, P52292, Q7Z3Z5, Q13901, Q96FF1, Q14566, Q14397, O14757, Q10570, Q14297, Q8NDT2, A8K4S5, B2RU05, P62988, Q9BYG9, P42704, Q9Y5S4, Q13415, Q9BT08, Q9GZY0
**ribosome**	P62988, Q9BYG9, Q9NVS2, Q6S4P3, Q9P150
**plasma membrane**	Q8WZ74, O43520, P15311, P27487, O95255, P14550, P08133
**cytoplasm**	P07437, P09467, Q8N1A4, P60709, P48736, B2RBL3, Q4W4Y1, Q49AJ4, P15311, P38606, Q9BYX7, P51572, Q5HY54, P14902, P40925, Q9P1R8, P18669, Q13464, Q6S4P3, P60842, Q9BYG9, O00299, P10599, P14550, Q13415, O95613, P13796, Q2QD09, Q99570, Q59EL5, P27797, P04040, B7ZMF0, P61981, Q13014, Q9BRL5, P08670, P50990, Q6IQ15, Q8TE87, Q9P150, Q14397, Q12882, P62166, B4DNK3, Q8WZ74, P07205, P37837, P31323, Q15751, Q96I26, Q8WXB2, P07203, P62988, P62937, O75901, Q9Y2I6, O75874, P42338, P57737, Q16719, Q3YAC3, P26373
**Golgi apparatus**	O60934, P30086, P04040, P51795, Q15699, Q5SX88, O00461, Q9NS84, P27487, B5MC68, Q96CC6, P51572, Q5HY54, P62166, B4DNK3, Q13464, Q15751, P11717, Q59GU6, P15907, Q86VU3, O75901, Q5QPU5, P57737, Q3YAC3, Q14789, Q9H9S5, P14410
**cytoplasmic vesicle**	P07355, P31146, B4DNK3, Q8WZ74, P52566, P11717, P30086, P00488, Q4W4Y1, B3V8S3, O00461, P08133, P40121, O75901, P35663, P08238, Q8IVZ4, Q86TH3, P57737, Q3MIV8, Q06830, P63104
**microtubule organizing center**	B3KX42, O14757, Q9P1R8, Q59G59, Q13464, B7ZMF0, Q9UFG9, Q6PEY2, Q4W4Y1, Q9BYG9, Q9Y2I6, P15311, Q9BRL5, Q76KD6, Q7Z3Z5, O95613
**intracellular non-membrane-bounded organelle**	P46940, Q5VU64, B3KX42, P07437, P35527, B7Z7S9, Q9UFG9, P23528, P35249, Q4W4Y1, O00488, P23527, P02730, Q9H7Z5, P07737, O75691, P35663, P13645, P13647, P02533, P17661, Q5HY54, P04080, P06702, P31146, Q9P1R8, P04083, Q8NDT2, Q14297, P19012, Q13464, Q9Y2K3, Q6S4P3, P12883, Q2NKX8, Q562R1, Q5JXT4, P42704, Q86TH3, P61586, Q9GZY0, P13796, O14646, A8MW06, Q92905, P52566, P04075, O60934, B7ZMF0, Q9Y4G6, Q6ZPD6, Q5JV91, Q9UKN7, Q13901, P63104, P63313, Q92817, P62988, Q16658, P35908, P26373, Q14573, P04259, Q96KK5, Q5T8A7, Q59G59, P60709, Q5HY93, Q5JVZ5, Q6PEY2, Q15699, A8CDT9, Q14192, P15311, Q8IU65, A7E2F8, Q9UJC3, Q9BYX7, P04264, Q96FF1, Q13895, Q9NQT8, O14757, P33778, Q96T58, P68363, P08779, Q9BYG9, P26447, Q13415, O95613, P04792, Q14019, P30086, Q14692, Q16352, Q16555, P40121, P42285, A6NIV8, Q9BRL5, P08670, Q7Z3Z5, Q9Y4C8, Q9NQZ2, Q9P150, Q96P04, P62166, Q9NVS2, B3V8S3, Q9Y2I6, Q07283, Q76KD6, Q7Z3Y8, Q3MIV8, Q7Z3Y7, Q9Y623
**axon**	P62166, Q86TH3, P08670, Q59G59, P60709, P30086, P58546, Q16555

Supplemental Figure S2.

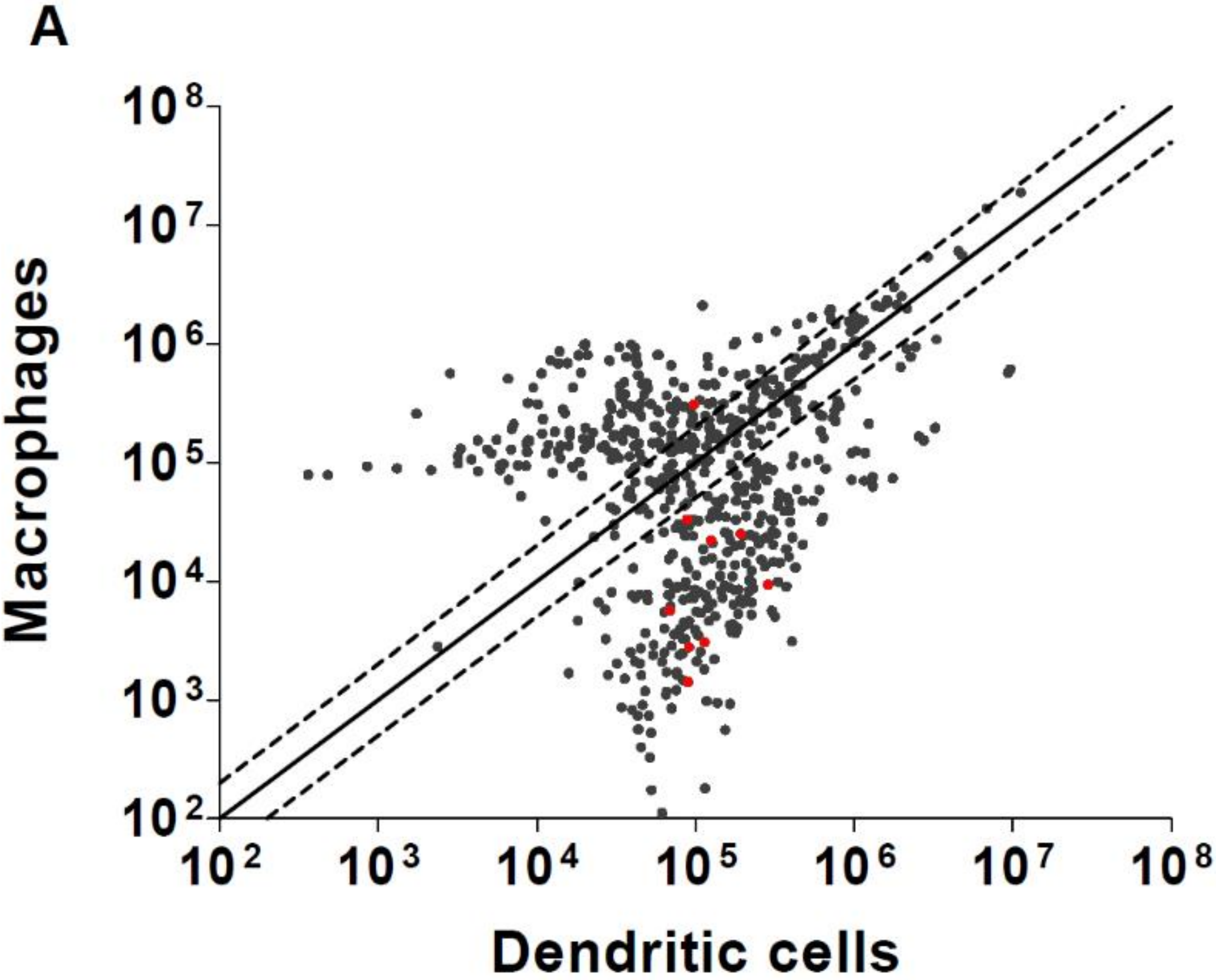

B

(Residue no.) FVIII peptide sequence	Average intensity Macrophages	Average intensity DCs	Ratio DCs over Macrophages
**(0373-0408) SVAKKHPKTWVHYIAAEEEDWDYAPLVLAPDDRSYK**	22240.7	125570.35	5.65
**(0459-0473) DTLLIIFKNQASRPY**	9438.75	288094.20	30.52
**(0459-0473) DTLLIIFKNQASRPY**	1422.90	89773.05	63.09
**(1126-1136) PKQLVSLGPEK**	311369.85	98318.25	0.32
**(1722-1740) AQSGSVPQFKKVVFQEFTD**	33374.65	89229.25	2.67
**(1768-1783) EDNIMVTFRNQASRPY**	3068.85	114580.00	37.34
**(1769-1783) DNIMVTFRNQASRPY**	2803.25	91587.90	32.67
**(2098-2111) ISQFIIMYSLDGKK**	25167.55	193812.35	7.70
**(2098-2112) ISQFIIMYSLDGKKW**	5674.20	69253.95	12.21
